# A study on current status and influencing factors of pregnant women’s body image in China: A mixed-methods study

**DOI:** 10.1186/s12884-026-09463-w

**Published:** 2026-06-26

**Authors:** YiFei Zhang, Rui Yu, Hong Zhao

**Affiliations:** 1 Department of General Surgery, the First Medical Center, Chinese PLA General Hospital, Beijing, China; 2 Peking Union Medical College Hospital, Chinese Academy of Medical Sciences & Peking Union Medical College, Beijing, China; 3 School of Nursing, Chinese Academy of Medical Sciences & Peking Union Medical College, No. 33, Badachu Road, Shijingshan District, Beijing, 100144 China

**Keywords:** Pregnant women, Body image, Influencing factors, Family support

## Abstract

**Background:**

Recent studies indicate that negative body image adversely affects the physical and psychological well-being of pregnant women and undermines family harmony, thereby jeopardizing maternal and infant health. Despite its significant impact, this issue has received scant scholarly attention in China. Body image is not formed in isolation; according to the socio-cultural model, it is constructed through the dynamic interplay of socio-cultural context, family interactions, media messages, and social comparisons. Therefore, it is imperative to investigate the current status and influencing factors of body image among pregnant women in China to establish an evidence base for developing culturally targeted interventions.

**Methods:**

An exploratory sequential mixed-methods design was employed. Phase 1 involved semi-structured in-depth interviews with 17 pregnant women, analyzed using reflexive thematic analysis to develop themes. Phase 2 consisted of a questionnaire survey with 160 pregnant women, utilizing a self-designed questionnaire, the Chinese version of the Body Image in Pregnancy Scale (BIPS), the Perceived Social Support Scale (PSSS), and the Simplified Coping Style Questionnaire (SCSQ). SPSS 25 was utilized for statistical analysis. The qualitative findings directly informed the focus and variable selection for the quantitative phase.

**Results:**

Three qualitative themes emerged: key factors influencing body image, consequences and outcomes of body image, and the desire for recognition and support. Quantitatively, body image dissatisfaction was moderate (BIPS score: 97.78 ± 15.57). Multiple linear regression identified greater gestational weight gain (β = 0.691, *p* = 0.008), lower family support (β = -0.804, *p* = 0.015), and parity (β = -6.819, *p* = 0.022) as significant predictors of poorer body image. Qualitative insights provided contextual depth to these statistical associations.

**Conclusions:**

Body image dissatisfaction was moderate among Chinese pregnant women and is influenced by weight gain, family support, and parity. The exploratory sequential design allowed for a deep understanding of lived experiences before quantitative measurement, ensuring the relevance of the measured constructs. Interventions should focus on weight management education and enhancing family support, particularly for first-time mothers, while also addressing and challenging the broader socio-cultural expectations surrounding unrealistic body ideals during pregnancy and the postpartum period.

**Supplementary Information:**

The online version contains supplementary material available at https://doi.org/10.1186/s12884-026-09463-w.

## Background

Pregnancy is a special stage in a woman’s life, accompanied by significant physiological and psychological changes. During this period, women’s perception of their body image is not only a matter of personal adaptation but also a deeply socio-culturally constructed process [1]. The socio-cultural model posits that body image is not formed in isolation; rather, it results from individuals’ continuous interaction with their socio-cultural environment—including family, peers, media, and social norms—through which they internalize external information, engage in comparisons, and develop self-evaluations [2].

In China, the unique collectivist culture, family-centric values, and increasingly globalized aesthetic ideals collectively form a complex backdrop against which pregnant women experience and evaluate their own bodies [3]. However, there is currently a lack of systematic research on pregnant women’s body image from a socio-cultural perspective in China. Therefore, this study aims to employ a mixed-methods approach within the theoretical framework of the socio-cultural model to investigate the current status of body image among Chinese pregnant women and identify its influencing factors, to provide empirical evidence for developing culturally targeted interventions.

Body image is a multi-dimensional concept that refers to the psychological picture of one’s own body formed by an individual, including cognition, attitude (such as emotion and evaluation) toward physiological and psychological functions, and influence on behavior [4]. Pregnancy is a special period for women. To adapt to the needs of fetal growth and development, with the participation of various hormones produced by the placenta, the pregnant woman’s body undergoes a series of adaptive anatomical and physiological changes to meet the needs of fetal growth and delivery [5]. As the fetus gradually grows, body changes become more pronounced, such as abdominal swelling, stretch marks, weight gain, edema, and melasma on the face [6]. In late pregnancy, physiological changes reach their peak, and pregnant women become more aware of these changes, which may gradually deviate from mainstream aesthetic standards [7]. In addition, pregnant women are emotionally fragile and sensitive during pregnancy, and their spouses’ and others’ views on their body image are more likely to contribute to negative body image [8].

Negative body image is prevalent among pregnant women. Roomruangwong et al. showed that 34.1% of pregnant women reported dissatisfaction with body image, and compared with the postpartum period, body image dissatisfaction during pregnancy was more severe [9]. Marshall et al. analyzed body image among pregnant women based on their Facebook posts [10]. The results showed that only 26% of pregnant women made positive comments about their body image, while 35% made negative comments [11, 12]. Some pregnant women regarded weight gain and body changes as negative signs and expressed a desire to quickly return to their pre-pregnancy body shape after childbirth [13]. Jawed-Wessel et al. found that nearly 28% of pregnant women expressed body image dissatisfaction [14].

Research to date indicates that factors influencing body image during pregnancy encompass media exposure, obstetric-related variables, and social support. Studies indicate that exposure to media content portraying idealized postpartum body shapes significantly reduces pregnant women’s body satisfaction, highlighting the importance of guiding them to critically evaluate media information [15, 16]. Support and encouragement from family and peers are equally crucial, as positive feedback helps enhance body satisfaction and emotional well-being. Furthermore, pre-pregnancy body mass index (BMI) and gestational weight gain are closely associated with body image satisfaction, with obesity or excessive weight gain often linked to lower body satisfaction [17, 18]. Current research on the relationship between maternal age and body image remains insufficient, though a few international studies suggest that adolescent pregnant women may face more severe body image concerns.

However, research on body image among pregnant women within the Chinese cultural context remains limited. Although body image disturbance is relatively common, prenatal healthcare personnel still pay insufficient attention to this psychological issue [19]. Some studies have pointed out that less than one-third of medical personnel assess body image among pregnant women during routine prenatal examinations. In addition, research on body image in China currently focuses on teenagers, college students, high school students, the elderly, or patients after cancer surgery, with less attention paid to pregnant women [20–22].

Therefore, it is necessary to understand the body image experiences of pregnant women in the context of Chinese culture, explore the factors that affect their body image, and subsequently provide solutions for interventions targeting negative body image among pregnant women.

## Methods

### Study design

**Fig. 1:**
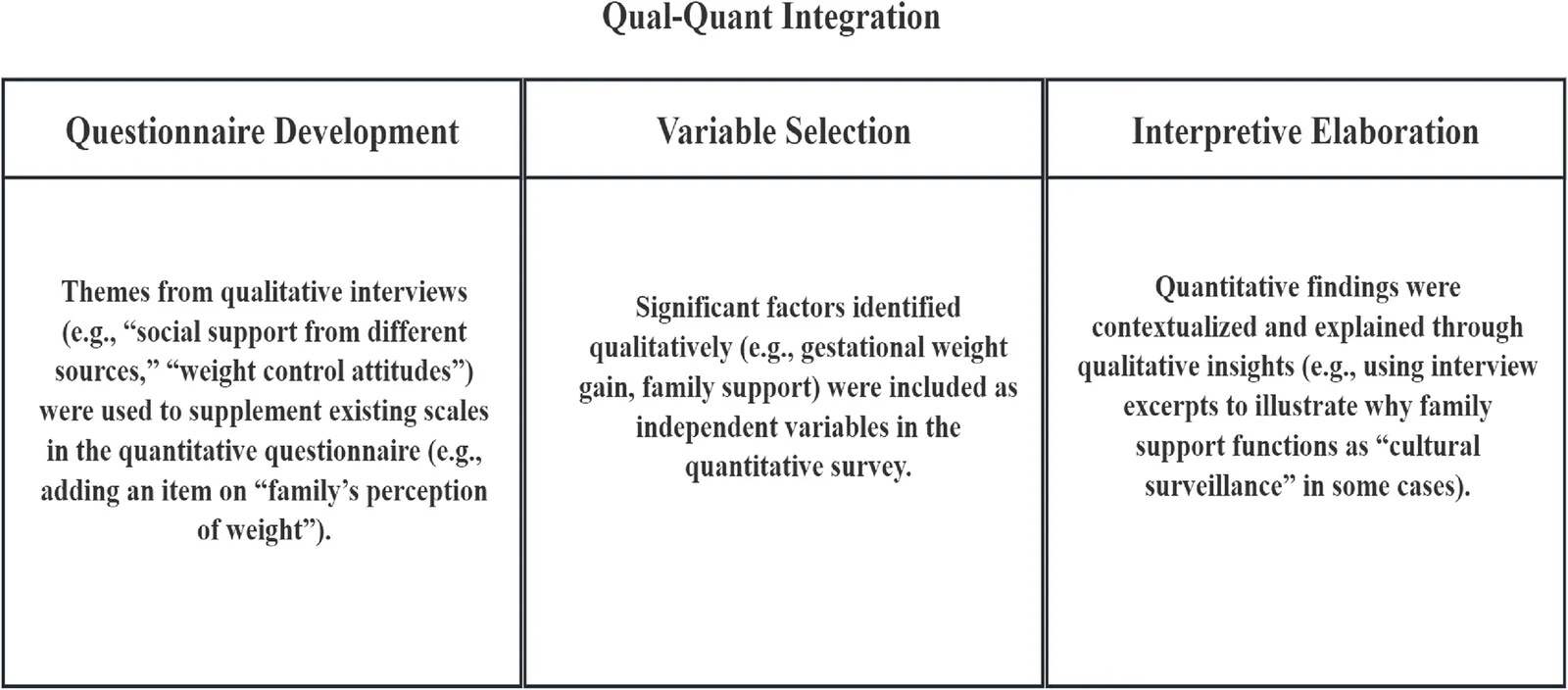
Qual-quant integration

An exploratory sequential mixed-methods design [23, 24] was used to investigate pregnant women’s body image in China. This study comprises two phases. In the first phase, qualitative research was conducted to explore the perception and cognition of body image among women in their third trimester through in-depth semi-structured interviews. In the second phase, the current status and influencing factors of pregnant women’s body image were investigated through a questionnaire survey. This questionnaire integrated themes and dimensions from the qualitative phase and was supplemented by items from existing scales. The rationale for this design was to first explore the lived experiences and subjective meanings of body image through qualitative interviews, and then to use these insights to inform and focus the subsequent quantitative survey. This approach ensured that the quantitative measurement was grounded in the real-world experiences of pregnant women, avoiding measurement of irrelevant or theoretical constructs (Fig. 1).

### Ethical approval and informed consent

This study was approved by the Ethics Committee, with the ethical approval number PUMCSON-2021-26. All participants voluntarily provided written informed consent after being fully informed of the research purpose, procedures, and potential risks. The research process strictly adhered to the ethical principles outlined in the Declaration of Helsinki, ensuring the protection of participants’ privacy, confidentiality, and autonomy.

### Qualitative phase—in-depth interview

#### Study design and setting

Qualitative data were collected through semi-structured in-depth interviews between January 1, 2022, and February 28, 2022, at Fengtai District Maternal and Child Health Care Hospital. The sample size was determined based on the data saturation principle, which means that sample recruitment continued until no new codes emerged. Ultimately, a total of 17 participants were enrolled. Eligibility criteria were defined as follows. Inclusion criteria comprised: (1) age > 18 years, (2) gestational age ≥ 28 weeks, and (3) provision of informed consent for voluntary participation. Exclusion criteria included: (1) diagnosed mental illness, (2) language or communication impairment, and (3) inability to independently complete the questionnaire.

#### Data collection

Before the interviews began, a survey was conducted to collect basic personal information from all 17 participants.

The semi-structured interview instrument comprised five questions (Supplementary 1). The interview location was a quiet and clean reception room for pregnant women at the Fengtai District Maternal and Child Health Hospital’s obstetrics clinic, which ensured the privacy of the interviews. Two researchers were involved: one recorded environmental information, interviewees’ nonverbal communication, and facial expressions, while the other conducted the interviews with pregnant women. We used a digital recorder to record the interview content. On the same day, the audio was transcribed, and another researcher compared the recordings again. All information is well preserved for later reference and confirmation. This study conducted a total of 17 interviews, each lasting 15–30 min. After each interview, the researcher transcribed the interview content verbatim within 24 h, repeatedly listening to the recording and proofreading, and recording nonverbal information, totaling approximately 100,000 words.

#### Data analysis

The qualitative data from 17 interview transcripts were analyzed to facilitate data management and the iterative coding process. This study employed reflexive thematic analysis [25] to identify and interpret patterns of meaning, with particular attention to how cultural contexts shaped participants’ experiences. The analysis followed an iterative six-phase approach: (1) familiarization with the data through repeated reading and memo-writing; (2) generating initial codes with attention to semantic and latent meanings; (3) searching for themes by organizing codes into potential patterns; (4) reviewing and refining themes through constant comparison with the dataset; (5) defining and naming themes with a focus on their conceptual significance within the Chinese sociocultural context; and (6) producing the final analysis. Throughout this process, researcher reflexivity was maintained through regular discussion and memoing to critically examine how pre-understandings and cultural positioning influenced theme development. The detailed analytical procedure is provided in Supplementary 2.

### Quantitative phase-questionnaire survey

#### Study design and setting

The quantitative phase is a cross-sectional study conducted through questionnaire surveys. Building on the three themes extracted from the qualitative phase, we explicitly operationalized qualitative insights into the quantitative survey design to ensure meaningful integration. For instance, the qualitative theme “Social support from different sources” revealed that family comments could act as either “cultural validation” or “cultural surveillance.” To capture this nuanced dynamic quantitatively, we retained the standardized family support subscale (PSSS) to measure overall perceived support, and supplemented it with a purpose-designed item in the demographic questionnaire: “What do your family members think about your weight?” This allowed us to triangulate the pervasive influence of familial attention identified qualitatively with a quantifiable behavioral frequency. Similarly, themes regarding weight control and attitudes informed our focus on gestational weight gain and pre-pregnancy BMI. During this phase, we recruited pregnant women in their third trimester who had initiated perinatal care at the antenatal clinic of Peking Union Medical College Hospital using a convenience sampling method from March 15, 2022, to September 30, 2022. The inclusion and exclusion criteria for participants remained consistent with those applied in the preceding qualitative phase.

#### Sample

During the qualitative phase, we identified social support from different sources, gestational weight gain, attitudes toward gestational weight gain, and emotional coping styles as prominent influencing factors of pregnant women’s body image within the Chinese cultural context. These factors were subsequently included as independent variables in this study.

Based on the qualitative findings and a review of the literature, a total of 14 variables were included for subsequent analysis. The sample size for studies examining influencing factors should be determined according to the requirements of statistical analysis; it is typically recommended to be at least 5 to 10 times the number of variables. Considering a 20% anticipated attrition rate, the final sample size was set at 170 participants.

#### Data collection

Data were collected electronically between March 15, 2022, and September 30, 2022, using a battery of instruments: a self-designed questionnaire (informed by qualitative findings and including items to capture relevant themes), the Chinese version of the Body Image in Pregnancy Scale (BIPS), the Perceived Social Support Scale (PSSS), and the Simplified Coping Style Questionnaire (SCSQ) (Supplementary 3: The Questionnaires and Measurement Scales).

The self-designed questionnaire was used to collect demographic data: age, height, weight, occupation, per capita family income, education level, and media usage time of pregnant women. Clinical data collected included gestational age, pregnancy frequency, parity, and conception method.

The Body Image in Pregnancy Scale (BIPS) was administered using the Chinese version translated by Sun Weijia [26–28]. Total scores on the BIPS range from 35 to 175, with higher scores indicating a more negative body image. The scale comprises 35 items across nine dimensions: dissatisfaction with body parts, perceived physical condition, appearance concern, dissatisfaction with facial features, perceived attractiveness to the opposite sex, appearance-related weight control, prioritization of appearance over bodily function, appearance-related avoidance behavior, and dissatisfaction with physiological changes during pregnancy.Items are rated on a 5-point Likert scale, with response options varying by section. The first section assesses perceptions of physical aspects during pregnancy (1 = strongly disagree to 5 = strongly agree); the second measures feelings about physical changes (1 = very satisfied to 5 = very dissatisfied); and the third evaluates appearance-related behaviors (1 = never to 5 = always). Items 9, 10, and 11 are reverse-scored. The scoring rate was calculated using the formula: (total score / maximum possible score) × 100%. The scale demonstrated good reliability, with a Cronbach’s α coefficient of 0.86 and a test-retest reliability of 0.99.

The Perceived Social Support Scale (PSSS), originally developed by Zimet [29, 30], was employed to assess participants’ perceived social support. Total scores on the PSSS range from 12 to 84, with scores of 12–36 indicating low perceived support, 37–60 indicating moderate support, and 61–84 indicating high support. The scale comprises 12 items measuring perceived social support across three dimensions: family support, friend support, and support from significant others. Each item is rated on a 7-point Likert scale (1 = very strongly disagree to 7 = very strongly agree). The PSSS is widely used internationally and demonstrated good reliability in the present study (Cronbach’s α = 0.91; test-retest reliability = 0.85).

The Simple Coping Style Questionnaire (SCSQ), developed by Xie Yaning [31, 32] within the Chinese cultural context, was administered to evaluate coping strategies. The SCSQ comprises two dimensions: positive coping and negative coping. The positive coping dimension score ranges from 0 to 24, with higher scores indicating a greater tendency to employ positive coping strategies. The negative coping dimension score ranges from 0 to 36, with higher scores reflecting a greater inclination toward negative coping strategies.The 20-item scale consists of the two dimensions: positive coping (8 items) and negative coping (12 items). Each item is rated on a 4-point Likert scale (0 = never, 1 = occasionally, 2 = sometimes, 3 = frequently). Respondents are classified as primarily adopting a positive coping style if their positive coping score exceeds their negative coping score, and vice versa. In this study, the scale demonstrated good reliability, with a Cronbach’s α of 0.90 for the overall scale, 0.89 for the positive coping dimension, and 0.78 for the negative coping dimension.

#### Data analysis

Data were analyzed using SPSS version 25.0. Continuous variables conforming to a normal distribution were summarized as mean ± standard deviation; otherwise, median and interquartile range were reported. Categorical variables, such as ethnicity, income level, and education level, were described using frequencies and percentages. Scale scores—including the Body Image in Pregnancy Scale, the Perceived Social Support Scale, and the Simplified Coping Style Questionnaire—were presented as mean ± standard deviation.

To identify factors influencing body image among pregnant women, univariate analyses of body image scores were first performed using independent t-tests and one-way analysis of variance (ANOVA), and relationships among continuous variables were examined via Pearson correlation analysis. Variables showing statistical significance in these analyses were then included as independent variables, with body image scores as the dependent variable, in a stepwise multiple linear regression model to identify independent predictors.

## Results

### Qualitative findings

A total of 17 participants were interviewed, with interview durations ranging from 15 to 30 min. They were assigned identifiers as F1 through F17. Participants were between 26 and 37 years of age, with gestational ages ranging from 32 to 39 weeks. Among the participants, 35.3% were multiparous women and 70.6% held a bachelor’s degree. See Table 1 for details.

**Table 1:** Demographic information of the interviewees (*n* = 17)

Number	Age	Pregnancy Week	Education level	Parity
F1	32	37	Bachelor degree	1
F2	35	38	Bachelor degree	0
F3	26	38	Bachelor degree	0
F4	30	36	High School Diploma	0
F5	31	39	Associate degree	1
F6	29	32	Bachelor degree	0
F7	34	37	Bachelor degree	0
F8	28	38	Bachelor degree	0
F9	33	36	Associate degree	2
F10	30	38	Bachelor degree	0
F11	32	35	Bachelor degree	0
F12	30	34	Associate degree	0
F13	37	34	Bachelor degree	1
F14	35	37	Bachelor degree	0
F15	33	37	Bachelor degree	1
F16	30	32	Associate degree	0
F17	28	32	Bachelor degree	0

Based on the results of the interviews, a total of 3 themes and 8 sub-themes were identified.

### Theme 1: Key factors influencing body image

This theme encompasses the primary factors that shape body image perceptions during late pregnancy. Our analysis highlights that these factors are not merely personal but are interpreted and experienced through the lens of China’s family-centered values and collectivist norms.

#### Sub-theme1-Social support from different sources

Pregnancy was described as a period of heightened need for support. Within the framework of China’s “family-centered childcare” model, a pregnant woman’s body often becomes a site of familial attention and commentary [33–35]. Positive feedback, especially from spouses affirming their continued affection despite physical changes, was experienced as validating and significantly boosted body satisfaction. In contrast, criticism—particularly from mothers-in-law—was frequently interpreted not as a casual remark but as a judgment on the woman’s adherence to maternal and feminine ideals, leading to pronounced distress. This distinction led us to interpret social support here not merely as assistance, but as a critical channel of cultural validation or surveillance.

- F14: “When I had severe eczema and felt terrible, my husband kept telling me he didn’t mind and that our health was all that mattered. His attitude really helped me accept myself.”
- F2: “My mother-in-law often points out how much weight I’ve gained. It brings me down and makes me wonder if I really am that heavy… it feels hurtful and even made me think about dieting.”

#### Sub-theme2-Emotional coping styles

Participants universally acknowledged increased emotional vulnerability. While recognizing the benefits of open expression, many described a tension with the Confucian-inspired norm of emotional restraint valued in Chinese culture [3]. Women who actively sought to confide in trusted others (e.g., spouses, colleagues) reported that sharing worries helped them normalize concerns and regain a positive outlook. Conversely, those who internalized their struggles described being trapped in a cycle of negative rumination, unable to adapt to bodily changes. This pattern led us to conceptualize coping styles as not just personal traits, but as situated practices navigating cultural expectations of feminine composure.

- F7: “Talking to my coworkers about my worries over gaining weight helped calm my mind. After talking, many of those fears just faded.”
- F1: “I see the stretch marks and weight gain, but I don’t like talking to others about these things. I end up digesting these emotions alone and struggling to accept the changes.”

#### Sub-theme3-Weight control during pregnancy

Weight control was a salient concern, framed by awareness of rising obesity rates and medical guidelines [36]. Successful management, often achieved through dietary control and prenatal yoga, was a major source of pride and body satisfaction. Conversely, perceived failure to control weight was linked to strong dissatisfaction and anxiety about health and social appearance. Our reflexive discussion highlighted that weight management in this context transcends personal health; it becomes a performance of self-discipline and responsibility toward the family lineage.

- F6: “I’ve been very careful with my diet and do yoga regularly. I’ve only gained weight from the baby’s growth, so I’m quite satisfied with my figure.”
- F9: “My weight has gone up a lot with each pregnancy. It’s hard to find clothes, and I even have a fatty liver now. It’s really worrying.”

#### Sub-theme4-Attitude towards weight gain during pregnancy

All participants experienced physiological weight gain, but their interpretations varied profoundly. Some reframed this change through a lens of natural process and temporary sacrifice, viewing their fuller figures as a “special kind of beauty” integral to the pregnancy journey, leading to higher body acceptance. Others interpreted rapid or significant gain as a personal loss of control and a deviation from their pre-pregnancy identity, resulting in negative self-evaluation. This sub-theme reveals how the same physiological event is constructed as either a culturally valorized maternal transformation or an individual shortcoming.

- F8: “People say I look fuller, and it’s true. But I see it as part of a natural, special process. This state has its own beauty—you accept it now and recover later.”
- F7: “I gained weight so quickly. I’ve never been this heavy before, and it makes me quite unhappy and worried.”

### Theme 2: Consequences and outcomes of body image

This theme elucidates how the body image perceptions formed by the factors above actively shape women’s well-being and actions in late pregnancy. The impact was found to be particularly acute within a cultural context that highly values maternal serenity and family-oriented sacrifice.

#### Sub-theme5-Emotional and psychological impact: conflict with the “ideal mother”

Body image directly influenced emotional states. Negative body image was reported to hinder the psychological transition to motherhood, fueling anxiety, sadness, and in some cases, transient resentment toward the pregnancy itself. We interpreted these emotional struggles as particularly distressing because they conflict with deep-seated cultural ideals of the serene, resilient, and self-sacrificing “good mother.” This internal conflict between feeling and expectation amplifies psychological distress.

- F10: “Seeing the stretch marks and darkening skin made me so sad and anxious about getting my body back. I cried once. My husband’s comfort later helped.”
- F7 (on early pregnancy): “The morning sickness was so bad it made me resent the baby for a while.”

#### Sub-theme6-Impact on health behavior intentions and decisions

Body image perceptions directly influenced concrete plans for postpartum health behaviors. Regarding breastfeeding, the cultural imperative to breastfeed (“everything for the child”) was complicated by body image anxieties, leading some to plan for shorter duration or conditional feeding to mitigate perceived bodily damage. In diet and exercise, body image acted as a primary motivator, driving strict regimens for some, while leading others toward potentially counterproductive practices like restrictive dieting. These findings show how body image becomes a key factor in navigating between cultural mandates of maternal duty and personal bodily integrity.

Postpartum Breastfeeding Decisions: Under the traditional parenting notion that “everything is for the child,” breastfeeding is often viewed as a fundamental maternal responsibility. However, body image anxiety complicates this obligation.

- F1: “I’ll pay more attention to managing my figure after birth. If I breastfeed, I can’t diet, so I plan to wean when I return to work.”
- F5: “I will breastfeed for over a year. I’ve heard it helps with postpartum recovery and weight loss.”

Diet and Exercise Behaviors: Body image perceptions actively shape daily health practices.

F6 engaged in strict dietary control and frequent yoga to manage weight. Conversely, F9 resorted to ineffective dieting attempts, and F10 needed to increase food intake due to being underweight, as advised by her doctor.

### Theme 3: The desire for recognition and support

A prominent, cross-cutting finding was the women’s expressed desire for their subjective bodily experiences and challenges to be fully acknowledged. This call for recognition critiques the implicit cultural tendency to reduce the pregnant woman to a vessel for fetal development.

#### Sub-theme7-Craving for emotional and attentive support

Participants desired emotional support that recognized them as whole persons, not just incubators. They longed for family members, especially partners, to attend to their development, nutrition, and appearance—their well-being as individuals—and not just the health of the fetus. This desire highlights a tension between traditional familial focus on the lineage and contemporary expectations for individual recognition within relationships.

- F7: “I hope my family would care about me—my development, my nutrition, my looks—not just the baby. That would make me feel truly cared for.”
- F11: “The hip pain at night is hard. My husband helps when he can, but he works. Sometimes I just feel helpless dealing with it alone.”

#### Sub-theme8-Seeking authoritative, culturally-nuanced professional guidance

Faced with a marketplace of conflicting information (traditional practices like “sitting the month” versus modern health advice), participants expressed a strong preference for professional guidance from healthcare providers [37]. They sought evidence-based, yet culturally sensitive, advice on body image management, postpartum recovery, and balancing maternal with fetal health. This trust in medical authority reflects a desire for a reliable navigational tool amidst competing cultural and commercial discourses on the pregnant body.

- F2 & F8: Expressed wanting professional advice on gentle body shaping and stretch mark prevention, valuing doctors’ opinions over commercial sources.
- F11: “I’m anxious about changes to my pelvic floor and muscles after birth. I wish doctors would tell us about recovery methods beforehand to ease that worry.”

### Quantitative results

#### Description of the sample

A total of 160 pregnant women participated in the survey. The mean age of participants was 33.43 ± 3.61 years. A high proportion were overweight or obese before pregnancy (71.9%). Detailed information is provided in Table 2.

The total body image score among pregnant women was 97.78 ± 15.57. The nine dimension scores were as follows: attention to body parts, 19.80 ± 5.29; perceived physical strength, 12.18 ± 4.06; appearance-related attention, 12.82 ± 3.34; evaluation of facial features, 10.13 ± 2.93; perceived attractiveness to the opposite sex, 11.18 ± 1.90; weight control motivated by appearance, 6.49 ± 1.71; prioritization of appearance over bodily function, 9.66 ± 2.18; acceptance of physiological changes during pregnancy, 5.19 ± 1.81; and appearance-related avoidance behavior, 5.77 ± 1.87.

The total perceived social support score among pregnant women was 66.39 ± 10.84. The subscale scores were as follows: family support 24.02 ± 3.63, friend support 22.12 ± 4.25, and other support 20.24 ± 4.87. The positive coping dimension score was 26.38 ± 5.76, and the negative coping dimension score was 10.53 ± 4.29. All participants demonstrated a positive coping style. Drawing on the qualitative data, we elaborate on these points in the discussion that follows. The participants’ demographic information, obstetric data, scores on the Perceived Social Support Scale (PSSS), and scores on the Simplified Coping Style Questionnaire (SCSQ) can be found in Supplementary 4.

#### Influencing factors of pregnant women’s body image

Univariate analysis revealed significant differences in pregnant women’s body image scores based on gestational weight gain (*p* = 0.011), parity (*p* = 0.046), and family support (*p* = 0.008). The results are shown in Table 2. Additionally, taking the total body image score as the dependent variable and the factors identified as statistically significant in the univariate analysis as independent variables, a Pearson correlation analysis was conducted. The results showed that the total body image score was significantly correlated with gestational weight gain (*r* = 0.235), family support score (*r* = -0.183), and parity (*r* = -0.158). Specifically, there was a significant positive correlation with gestational weight gain (*p* < 0.01), indicating that greater weight gain during pregnancy was associated with higher body image scores. In contrast, there was a significant negative correlation with family support score (*p* < 0.05), suggesting that higher levels of family support were associated with lower body image scores. Additionally, a significant negative correlation was observed with parity, meaning that more childbirth experiences were linked to lower body image scores. The results are shown in Table 3.

**Table 2:** Univariate analysis of body image in pregnant women (*n* = 160)

Variable	Group	Number	Constituent Ratio (%)	BIPS scores	F/t	*P*
Age	≤35	115	71.8	97.36 ± 15.34	0.376^a^	0.770
	>35	45	28.2	100.56 ± 9.20		
Nation	Han	148	92.5	98.33 ± 15.43	1.595^a^	0.113
	Minorities	12	7.5	90.92 ± 16.35		
Education level	Associate degree or below	7	4.4	103.14 ± 20.91	1.092^b^	0.433
	Undergraduate	66	41.3	98.83 ± 16.20		
	Postgraduate	87	54.3	96.54 ± 14.65		
Homeplace	Town	150	93.8	97.89 ± 15.53	0.371^a^	0.711
	Rural	10	6.2	96.00 ± 16.90		
Religious beliefs	Yes	11	6.9	97.36 ± 18.99	-0.091^a^	0.928
	No	149	93.1	97.81 ± 15.36		
Occupation	Company Employees	97	60.6	98.29 ± 15.96	0.371^b^	0.868
	National Civil Servants	17	10.6	95.47 ± 13.26		
	Teacher	13	8.2	98.46 ± 14.26		
	Housewives	5	3.1	104.60 ± 16.10		
	Doing Business	3	1.9	95.33 ± 8.15		
	Others	25	15.6	95.92 ± 17.33		
Income	<5000 RMB	2	1.3	122.00 ± 2.83	1.807^b^	0.148
	5000–9999 RMB	21	13.1	95.71 ± 18.67		
	10,000–15,000 RMB	42	26.3	97.02 ± 17.96		
	>15,000 RMB	95	59.3	98.05 ± 13.46		
Pregnant weight gain	<10 KG	73	45.6	94.15 ± 14.39	4.649^b^	0.011*
	11-20KG	82	51.2	100.29 ± 15.77		
	>20KG	5	3.2	109.40 ± 18.16		
Prepregnancy BMI	Normal	45	28.1	96.02 ± 15.44	0.445^b^	0.642
	Overweight	81	50.6	98.16 ± 15.55		
	Obesity	34	21.3	99.18 ± 16.03		
Cohabitants	Spouses	148	92.5	97.41 ± 15.00	0.807^b^	0.448
	Parents/Parents-in-law	9	5.6	100.33 ± 24.15		
	Others	3	1.9	108.00 ± 13.89		
The relationship with the husband	Generally	3	1.9	106.33 ± 20.98	0.460^b^	0.632
	Intimate	34	21.3	97.50 ± 15.09		
	Very Intimate	123	76.8	97.64 ± 15.66		
Self-perceived weight	Light	4	2.5	104.00 ± 8.68	2.205^b^	0.114
	Normal	93	58.1	95.65 ± 14.59		
	Overweight	63	39.4	100.52 ± 16.89		
Family members perceive your weight	Light	15	9.4	96.07 ± 16.66	0.375^b^	0.688
	Normal	112	70.0	97.43 ± 14.93		
	Overweight	33	20.6	99.73 ± 17.43		
Social media usage time	<1 h per day	88	55.0	97.93 ± 16.20	0.351^b^	0.788
	1–3 h per day	60	37.4	97.65 ± 15.00		
	3–5 h per day	10	6.3	99.30 ± 14.76		
	>5 h per day	2	1.3	87.00 ± 14.14		
The degree of influence of social media	Not Affected	57	35.6	94.11 ± 15.99	2.732^b^	0.068
	Somewhat affected	86	53.8	99.36 ± 14.00		
	Significant Impact	18	10.6	102.06 ± 19.79		
Gestational age	Under 37 weeks	68	42.5	99.09 ± 15.64	0.917^a^	0.361
	37 weeks and above	92	57.5	96.80 ± 16.53		
Pregnancy frequency	Once	83	51.9	97.52 ± 14.67	0.265^b^	0.767
	2 times	47	29.4	99.04 ± 15.89		
	3 or more times	19	18.7	96.50 ± 17.76		
Parity	0 times	124	77.5	99.10 ± 14.73	2.738^b^	0.046*
	Once	33	20.6	91.79 ± 16.96		
	2 or more times	3	1.9	104.00 ± 26.87		
History of miscarriage	0 times	99	61.9	97.64 ± 15.03	0.019^b^	0.981
	Once	46	28.8	97.85 ± 15.87		
	2 or more times	15	9.4	98.47 ± 18.97		
Assisted reproductive technology (ART) pregnancy	No	134	83.7	98.52 ± 15.13	1.383^a^	0.169
	Yes	26	16.3	93.92 ± 17.46		
Pregnancy mode	Planned	132	82.5	97.43 ± 15.63	-0.604^a^	0.547
	Unexpected Pregnancy	28	17.5	99.39 ± 15.46		
Family support	≤ 15	1	0.63	147.00	4.099^b^	0.008**
	16–20	32	20.00	100.28 ± 11.90		
	21–25	63	39.38	97.44 ± 16.43		
	≥ 26	64	40.00	97.78 ± 15.57		
Friends support	≤ 15	4	2.50	85.50 ± 24.58	1.880^b^	0.135
	16–20	54	33.75	99.50 ± 15.72		
	21–25	63	39.38	99.19 ± 15.30		
	≥ 26	39	24.38	94.36 ± 14.29		
Other support	≤ 15	18	11.25	99.94 ± 22.43	0.973^b^	0.407
	16–20	66	41.25	99.73 ± 14.96		
	21–25	47	29.38	95.49 ± 13.83		
	≥ 26	29	18.13	95.69 ± 14.55		
Perceived social support	Moderate level	50	31.25	99.86 ± 16.28	1.307	0.255
	Advanced level	110	68.75	96.83 ± 16.21		
Positive dimension	≤ 15	6	3.75	97.50 ± 9.89	0.880^b^	0.477
	16–20	16	10.00	98.88 ± 15.46		
	21–25	58	36.25	100.43 ± 13.96		
	26–30	37	23.13	94.68 ± 18.03		
	>30	43	26.88	96.49 ± 16.02		
Negative dimension	≤ 5	19	11.88	92.89 ± 15.29	0.690^b^	0.600
	6–10	68	42.50	97.53 ± 13.46		
	11–15	46	28.75	98.85 ± 14.05		
	16–20	24	15.00	100.38 ± 23.26		
	>20	3	1.88	97.00 ± 9.64		

^a^ represents independent sample t-test

^b^ represents ANOVA test

* *P* < 0.05, **P < 0.01 indicate statistically significant difference

**Table 3:** Correlation analysis of pregnant women’s body image with gestational weight gain, parity, and family support

	Weight gain during pregnancy	Parity	Family support	Body image of pregnant women
Weight gain during pregnancy	1			
Parity	-0.208	1		
Family support	-0.015	-0.106	1	
Body image of pregnant women	0.235**	-0.158*	-0.183*	1

*P < 0.05, **P < 0.01 indicate statistical significance

In the multiple linear regression analysis, weight gain during pregnancy, family support, and parity were factors affecting the body image of pregnant women. The assignment of independent variables is shown in Table 4. The results are shown in Table 5.

**Table 4:** Assignment of independent variables

Independent Variables	Method Of Assigning
Parity	Dummy variables were created with ‘0 times’ as the reference category, 0 Times (Z1 = 0, Z2 = 0, Z3 = 0), 1 Time (Z1 = 0, Z2 = 1, Z3 = 0), 2 Times Or More (Z1 = 0, Z2 = 0, Z3 = 1)
Weight Gain During Pregnancy	Original Value Input
Family Support Score	Original Value Input

**Table 5:** Multiple linear regression analysis of factors influencing body image in pregnant women (*n* = 160)

Independent Variable	β	Standard error	β’	t	*P*	VIF
Constant	110.714	8.722	-	12.693	0.000	
Weight Gain During Pregnancy	0.691	0.259	0.204	2.672	0.008**	1.032
Family Support	-0.804	0.328	-0.187	-2.449	0.015*	1.035
Parity	-6.819	2.947	-0.178	-2.314	0.022*	1.043

R^2^ = 0.128, adjusted R^2^ = 0.100, F = 4.532, *P < 0.05, **P < 0.01 indicate statistical significance

## Discussion

This study employed an exploratory sequential mixed-methods design to investigate the current status and influencing factors of body image among pregnant women in China (primarily urban). By integrating qualitative narratives with quantitative measurements, the research not only identified significant predictors of body image dissatisfaction—gestational weight gain, family support, and parity—but also elucidated the profound cultural meanings and lived experiences underlying these variable relationships. The following discussion interprets the findings within the framework of the sociocultural model and compares them with the existing literature.

### Gestational weight gain: conflict between physiological reality and cultural scripts

Based on the initial qualitative phase, we identified gestational weight gain as a potential factor influencing pregnant women’s body image. The quantitative result showed that gestational weight gain was a significant positive predictor of body image dissatisfaction (β = 0.691, *p* = 0.008). This finding aligns with a substantial body of international research. For example, a prospective study by Hill et al. (2013) confirmed a positive correlation between gestational weight gain and body dissatisfaction [13]. Linde et al. (2022) also indicated that a higher body mass index in late pregnancy is associated with more negative body image [38]. Our quantitative results support this general association.

However, the qualitative themes of “Weight Control during Pregnancy” and “Attitude toward Weight Gain” reveal the unique manifestation of this association within the Chinese cultural context. Some participants reframed weight gain as a “special kind of beauty” and a natural process, which is similar to the finding by Watson et al. (2016) that some pregnant women can “normalize” bodily changes [39]. Yet, more participants exhibited anxiety and a sense of loss of control due to weight gain, consistent with the conclusion in the study by Shiraishi et al. (2024) that “weight concern” is a core stressor [18]. The qualitative analysis of this study further elucidates that, within the Chinese context, such anxiety stems not only from comparisons with one’s pre-pregnancy body but is also rooted in two distinct cultural-psychological mechanisms. First, guilt over “failing to adhere to medical guidelines” ^6,7^ reflects how, in a society where medical authority is highly respected, gestational weight management has been intensely medicalized into a performance of responsible “qualified motherhood.” Second, fear of “deviating from mainstream aesthetic standards” is closely tied to China’s long-standing collectivist culture, which emphasizes external evaluation and social conformity.

It must be noted that the sample in this study was predominantly drawn from high-level hospitals in Beijing, with participants generally possessing higher education levels and socioeconomic status. This demographic may be more likely to access and internalize scientific knowledge on gestational weight management, while simultaneously being more exposed to urban middle-class aesthetic ideals. Consequently, the observed “pressure for medical guideline compliance” and “anxiety over mainstream aesthetics” may be particularly pronounced within this group. Future research should include pregnant women from broader socioeconomic backgrounds to examine the generalizability of these cultural-psychological mechanisms across different populations—especially among rural and migrant women, who may have lower access to healthcare resources or be more influenced by traditional beliefs such as “nourishing the fetus without gaining maternal weight.”

### Family support and emotional coping: resources and negotiation within relational networks

During the qualitative phase, we found that family support—from spouses and mothers-in-law—is a significant factor influencing body image among pregnant women in China, where family values are deeply emphasized. The quantitative result found family support to be a significant negative predictor of body image dissatisfaction (β = -0.804, *p* = 0.015). This is consistent with the direction of research by Cho et al. (2022) on the protective role of social support against postpartum depression [40], and directly extends the role of support networks to the domain of pregnancy body image. The Multidimensional Scale of Perceived Social Support (PSSS) developed by Zimet et al. (1988) demonstrated good applicability in this study, with the family support subscale scoring the highest and having the strongest predictive power [29].

The important extension of this study lies in the qualitative analysis revealing the dual cultural role of family support in the Chinese context: spousal support as “cultural validation,” while comments from family members such as mothers-in-law may constitute “cultural surveillance.” This differs from Western research that more often focuses on the general positive effects of partner support [14] or peer influence [41]. For instance, Coyne et al. (2018) emphasized the influence of media and peer comparison [16, 41], whereas our study found that within China’s collectivist and family-centric culture, feedback from core family members carries heavier emotional and symbolic weight, and the pregnant woman’s body becomes a “site of familial attention and commentary.” This finding enriches the sociocultural model, emphasizing that in collectivist societies, the “sociocultural” dimension of the model should place greater emphasis on the norms and pressures carried by family systems, interpersonal relationships, and face-to-face interactions.

Furthermore, the qualitative theme “Emotional Coping Styles” reveals the dynamic negotiation between individual agency and prevailing cultural norms as pregnant women navigate body image-related stress. Participants commonly experienced a tension in emotional expression: recognizing the benefits of disclosure while simultaneously feeling the weight of traditional cultural expectations for emotional restraint. Women who actively sought emotional support from trusted others (e.g., spouses) were often better able to reframe their bodily perceptions; conversely, those who internalized their emotions were more prone to persistent distress. This aligns with the sociocultural model’s perspective that individual coping behaviors do not occur in a vacuum but are the result of negotiation between internalized social norms (such as the emotional composure expected of a “virtuous wife and good mother”) and accessible social resources (such as empathetic family support). A simplified “positive-negative” coping dichotomy may obscure the culturally situated nature of these strategies. Therefore, future interventions, in addition to providing family support education, could incorporate cognitive-behavioral training focused on emotional management and communication skills during pregnancy. This would aim to help pregnant women utilize social support resources more effectively while respecting cultural norms of emotional expression.

It is important to contextualize these findings within the study’s limitations and the specific characteristics of Chinese families. The observed dual role of family support—as both validation and surveillance—is deeply embedded in China’s intergenerational cohabitation norms and the cultural script of “xiaoshun” (filial piety). In many Chinese households, especially where multigenerational living is common, a pregnant woman’s body is not merely a private concern but becomes a legitimate focus of familial discourse, often involving mothers-in-law whose comments carry the weight of generational authority and traditional wisdom. However, our sample primarily consisted of urban, well-educated women who may possess greater autonomy in negotiating these familial dynamics. The experience of pregnant women in more traditional or rural settings, where patriarchal structures and intergenerational hierarchies are stronger, might differ significantly. For instance, the “cultural surveillance” from family elders could be more pronounced and less easily challenged. Furthermore, the pressure to maintain emotional restraint and embody the ideal of a “virtuous wife and good mother” may be intensified in contexts with stronger adherence to traditional Confucian values. Future research should explore how socioeconomic status, regional differences (e.g., urban vs. rural), and specific family structures (e.g., nuclear vs. extended families) moderate the dual nature of family support and its impact on body image, thereby empowering pregnant women to navigate this duality without compromising their mental well-being or familial harmony.

### Parity: The mediation of maternal experience and cultural narratives

Quantitative results showed that multiparous women had significantly higher body image satisfaction. This finding engages in dialogue with and shows differences from some international studies. For example, the study by Roomruangwong et al. (2017) did not find a significant association between parity and body dissatisfaction [9]. Soyturk, A et al. (2025) indicated that adult pregnant women adapt better than adolescents [42]. The qualitative insights from this study offer a potential cultural explanation: In China, the identity of a mother of multiple children is often linked to positive cultural narratives such as “maturity,” “experience,” and “adding offspring to the family.” Therefore, multiparous women may more easily integrate bodily changes into this socioculturally endorsed narrative of “motherhood,” thereby reducing the conflict between self-body evaluation and the ideal image. This suggests that the protective effect of parity may be significantly moderated by cultural scripts, being more pronounced in societies that emphasize values such as having many children or mature motherhood.

Chinese society has long celebrated multiparity through deeply embedded cultural sayings such as “more children, more blessings” (duozi duofu) and “adding offspring to continue the family line” (tianding jinkou). These traditional proverbs frame having multiple children not merely as a personal choice but as a marker of familial prosperity, social continuity, and feminine virtue. Building on these culturally embedded narratives, multiparous women in our study appeared to draw on the positive identities of “maturity” and “experience” to reframe their bodily changes in ways that reduced potential body image concerns. Rather than viewing postpartum bodily transformations as flaws or deviations from youthful ideals, these women often interpreted them through the lens of maternal competence and embodied wisdom. As one participant (F9) articulated: “With my first pregnancy, I was anxious about every stretch mark and change. This time, I understand that these transformations are temporary and meaningful. I feel like my body knows what it’s doing—it’s part of becoming a mother, of gaining experience.” This narrative exemplifies how the identity of an “experienced mother” provides a positive interpretive framework through which bodily changes are understood as functional, purposeful, and even affirming, thereby reducing the dissonance between self-evaluation and internalized ideals of feminine beauty.

However, this interpretation must be considered alongside the study’s limitations and remains tentative rather than generalizable. Our sample consisted primarily of urban, well-educated women with higher socioeconomic status, for whom having multiple children may simultaneously signal financial stability, access to quality healthcare, and the ability to leverage resources—factors that could independently buffer body image distress regardless of parity. For these women, multiparity may represent an empowered choice enabled by privilege rather than a source of strain. In contrast, for women situated in different social strata—particularly those who experience multiparity under conditions of economic constraint, limited reproductive autonomy, or familial pressure to produce male offspring—the relationship between parity and body image may diverge substantially. In such contexts, cumulative pregnancies might exacerbate body dissatisfaction due to inadequate healthcare access, nutritional stress, or the symbolic weight of bearing children under compulsion rather than choice. Future research should therefore examine how cultural narratives, socioeconomic conditions, and evolving policy contexts—such as China’s shift from strict family planning to pronatalist incentives—intersect to shape the embodied experience of motherhood across diverse populations in contemporary China. Understanding these intersecting influences will be essential for developing culturally sensitive support systems that address the full spectrum of women’s reproductive experiences.

### Theoretical and practical implications

Beyond identifying specific predictors, this study offers a culturally situated extension to the sociocultural model of body image. While the original model emphasizes broad societal influences (e.g., mass media, peer comparison), our findings foreground the family system as the primary and affectively charged conduit through which cultural norms are transmitted and internalized within a collectivist context. We posit that in cultures like China, proximal, face-to-face interactions within the family carry more immediate weight for self-evaluation than distal media ideals, a refinement that future cross-cultural research should test.

Practically, this reconceptualization shifts intervention targets. Moving beyond generic health education, our findings advocate for culturally tailored protocols that: (1) implement “family support training” to equip partners and key relatives with skills for providing affirming support, and (2) develop “cognitive restructuring modules” that help pregnant women navigate the conflict between internalized aesthetic standards and the normative physiology of pregnancy. These interventions should be developed and evaluated within the specific sociocultural settings they intend to serve, rather than adopting Western models uncritically.

### Research limitations, comparisons, and future directions

While this study offers valuable insights, its limitations must be acknowledged to accurately interpret the findings and guide future scholarship.

The primary limitation lies in the sampling strategy. The quantitative sample (*n* = 160), drawn from a prestigious tertiary hospital in Beijing, represents a highly specific demographic: overwhelmingly urban (93.8%), highly educated (95.6% with bachelor’s degree or higher), and economically advantaged (85.6% with high household income). This limits the generalizability of the quantitative findings to the broader, socioeconomically diverse Chinese obstetric population, including rural, migrant, or less-educated women whose experiences and resources may differ drastically. The qualitative sample (*n* = 17) from a secondary hospital provided some contrast but was not purposefully designed for comparative analysis. Future research must prioritize stratified, multi-center, or nationwide sampling to capture the full spectrum of socioeconomic, regional, and cultural diversity within China. This will allow for meaningful subgroup analyses and more representative conclusions.

The cross-sectional design of the quantitative phase limits causal inferences. A longitudinal cohort study tracking women from early pregnancy through the postpartum period is essential to understand the temporal dynamics of body image. Such a design could determine whether body dissatisfaction predicts excessive gestational weight gain or vice versa, and how body image evolves after childbirth, including its impact on breastfeeding duration, postpartum mental health, and inter-pregnancy intervals.

Research should also expand to include the perspectives of partners and key family members. Furthermore, investigating the effectiveness of the culturally tailored interventions suggested above through randomized controlled trials would be a vital next step in translating knowledge into evidence-based practice.

## Conclusions

From the perspective of the socio-cultural model, body image dissatisfaction was moderate among Chinese pregnant women and is influenced by weight gain, family support, and parity. The exploratory sequential design allowed for a deep understanding of lived experiences before quantitative measurement, ensuring the relevance of the measured constructs. Interventions should focus on weight management education and enhancing family support, particularly for first-time mothers, while also addressing and challenging the broader socio-cultural expectations surrounding unrealistic body ideals during pregnancy and the postpartum period.

## Supplementary Information

Supplementary Material 1

Supplementary Material 2

Supplementary Material 3

Supplementary Material 4

## Data Availability

The data used for the current study are available from the the first author on reasonable request.

## Abbreviations

ART: Assisted Reproductive Technology
BIPS: Body Image in Pregnancy Scale
BMI: Body Mass Index
PSSS: Perceived Social Support Scale
SCSQ: Simplified Coping Style Questionnaire

## References

[1] Crossland AE, Munns L, Kirk E, Preston CEJ. Comparing body image dissatisfaction between pregnant women and non-pregnant women: a systematic review and meta-analysis. BMC Pregnancy Childbirth. 2023;23(1):709. Published 2023 Oct 4. 10.1186/s12884-023-05930-w PMC10548696 37794358

[2] Azevedo A, Azevedo ÂS. Implications of Socio-Cultural Pressure for a Thin Body Image on Avoidance of Social Interaction and on Corrective, Compensatory or Compulsive Shopping Behaviour. Int J Environ Res Public Health. 2023;20(4):3567. 10.3390/ijerph20043567. Published 2023 Feb 17. 36834261 PMC9959199

[3] Stojcic I, Dong X, Ren X. Body Image and Sociocultural Predictors of Body Image Dissatisfaction in Croatian and Chinese Women. Front Psychol. 2020;11:731. 10.3389/fpsyg.2020.00731. Published 2020 May 6. 32435214 PMC7218091

[4] Zhao F, Zhang X, Yue C, Zhang Z, Yuan M, Chen X. Association Between Body Image, Fear of Childbirth and Maternal-Fetal Attachment: The Relative Mediation Analysis. J Adv Nurs. 2025;81(10):6525–36. 10.1111/jan.16800. 39930321

[5] American College of Obstetricians and Gynecologists. ACOG Committee opinion 548: weight gain during pregnancy. Obstet Gynecol. 2013;121(1):210–2. 10.1097/01.aog.0000425668.87506.4c. 23262962

[6] Chen ML, Chang SR. The relationship between body dissatisfaction and postpartum depressive symptoms: A cross-sectional study. J Affect Disord. 2023;324:418–23. 10.1016/j.jad.2022.12.102. 36586599

[7] Wu Y, Yu S, Dai J, et al. Predictors of body image dissatisfaction among women at different stages of pregnancy:A cross-sectional study. Midwifery. 2024;129:103903. 10.1016/j.midw.2023.103903. 38056099

[8] Kadam KS, Anvekar AR, Unnithan VB. Depression, sleep quality, and body image disturbances among pregnant women in India: a cross-sectional study. J Yeungnam Med Sci. 2023;40(4):394–401. 10.12701/jyms.2023.00087. 37157779 PMC10626296

[9] Roomruangwong C, Kanchanatawan B, Sirivichayakul S, Maes M. High incidence of body image dissatisfaction in pregnancy and the postnatal period: Associations with depression, anxiety, body mass index and weight gain during pregnancy. Sex Reprod Healthc. 2017;13:103–9. 10.1016/j.srhc.2017.08.002. 28844350

[10] Marshall E, Moon MA, Mirchandani A, et al. Baby Wants Tacos: Analysis of Health-Related Facebook Posts from Young Pregnant Women. Matern Child Health J. 2019;23(10):1400–13. 10.1007/s10995-019-02776-7. 31222598 PMC7055721

[11] Figueiredo B, Tendais I, Dias CC. Maternal adjustment and maternal attitudes in adolescent and adult pregnant women. J Pediatr Adolesc Gynecol. 2014;27(4):194–201. 10.1016/j.jpag.2013.09.014. 24656707

[12] Goodwin A, Astbury J, McMeeken J. Body image and psychological well-being in pregnancy. A comparison of exercisers and non-exercisers. Aust N Z J Obstet Gynaecol. 2000;40(4):442–7. 10.1111/j.1479-828x.2000.tb01178.x. 11194433

[13] Hill B, Skouteris H, McCabe M, Fuller-Tyszkiewicz M. Body image and gestational weight gain: a prospective study. J Midwifery Womens Health. 2013;58(2):189–94. 10.1111/j.1542-2011.2012.00227.x. 23458678

[14] Jawed-Wessel S, Herbenick D, Schick V. The Relationship Between Body Image, Female Genital Self-Image, and Sexual Function Among First-Time Mothers. J Sex Marital Ther. 2017;43(7):618–32. 10.1080/0092623X.2016.1212443. 27420566

[15] Becker E, Rodgers RF, Zimmerman E. #Body goals or #Bopo? Exposure to pregnancy and post-partum related social media images: Effects on the body image and mood of women in the peri-pregnancy period. Body Image. 2022;42:1–10. 10.1016/j.bodyim.2022.04.010. 35594726

[16] Samra A, Dryer R. Problematic social media use and psychological distress in pregnancy: The mediating role of social comparisons and body dissatisfaction. J Affect Disord. 2024;361:702–11. 10.1016/j.jad.2024.06.057. 38897304

[17] Kuczyk C, Mbang Springer DL, Dickert JJ, et al. Association Between Body Image Profiles, Pre-pregnancy BMI and Weight Gain During Pregnancy. Obes Facts Published online January. 2026;9. 10.1159/000550432. PMC12890298 41511924

[18] Shiraishi M, Kurashima Y, Harada R. Association Between Body Image Before and During Pregnancy and Gestational Weight Gain in Japanese Women: A Prospective Cohort Study. Matern Child Health J. 2024;28(4):708–18. 10.1007/s10995-023-03854-7. 38051453 PMC10963548

[19] Plante AS, Doyon AA, Savard C, et al. Weight Changes and Body Image in Pregnant Women: A Challenge for Health Care Professionals. Can J Diet Pract Res. 2020;81(3):137–41. 10.3148/cjdpr-2020-007. 32072818

[20] Yuan Y, Hu J, Sun L et al. An association between body image dissatisfaction and digit ratio among Chinese children and adolescents. Sci Rep. 2021;11(1):5217. Published 2021 Mar 4. 10.1038/s41598-021-84711-x PMC7970844 33664410

[21] Xu W, Wan H, Xiang L, Wang S, Zhu Y, Zheng M. Effectiveness of acceptance commitment therapy for head and neck cancer patients with body image distress in China: a study protocol for randomised controlled trial. BMJ Open. 2024;14(9):e085551. 10.1136/bmjopen-2024-085551. Published 2024 Sep 5. 39242157 PMC11535697

[22] Xu W, Zhou L, Zhao C, Zhou Y, Chen S, Yang L. Evaluating Body Image Disturbance and Its Influencing Factors in Breast Cancer Patients Following Unilateral Mastectomy. Psychiatry Clin Psychopharmacol. 2024;34(4):328–35. 10.5152/pcp.2024.24953. 39772297 PMC11744373

[23] Bailey PK, Hole BD, Plumb LA, Caskey FJ. Mixed-methods research in nephrology. Kidney Int. 2022;101(5):895–905. 10.1016/j.kint.2022.01.027. 35227687

[24] Ismaila N, Harvey BE, Einhaus K, Mbuagbaw L, Ma J, Thabane L. Barriers and facilitators to implementing the living guideline development framework in oncology: a mixed methods study. BMJ Open. 2026;16(1):e109470. 10.1136/bmjopen-2025-109470. Published 2026 Jan 16. 41545044 PMC12815089

[25] Braun V, Clarke V. A critical review of the reporting of reflexive thematic analysis in Health Promotion International. Health Promot Int. 2024;39(3):daae049. 10.1093/heapro/daae049. 38805676 PMC11132294

[26] Watson B, Fuller-Tyszkiewicz M, Broadbent J, Skouteris H. Development and validation of a tailored measure of body image for pregnant women. Psychol Assess. 2017;29(11):1363–75. 10.1037/pas0000441. 28125247

[27] Sun W, Chen D, Wang J, Liu N, Zhang W. Physical activity and body image dissatisfaction among pregnant women: A systematic review and meta-analysis of cohort studies. Eur J Obstet Gynecol Reprod Biol. 2018;229:38–44. 10.1016/j.ejogrb.2018.07.021. 30099226

[28] Sun WJ. Translation and validation of the Body Image in Pregnancy Scale [master’s thesis]. Jilin: Jilin University; 2019.

[29] Zimet GD, Powell SS, Farley GK, Werkman S, Berkoff KA. Psychometric characteristics of the Multidimensional Scale of Perceived Social Support. J Pers Assess. 1990;55(3–4):610–7. 10.1080/00223891.1990.9674095. 2280326

[30] Xie B, Chou CP, Spruijt-Metz D, et al. Weight perception and weight-related sociocultural and behavioral factors in Chinese adolescents. Prev Med. 2006;42(3):229–34. 10.1016/j.ypmed.2005.12.013. 16458956

[31] Feng XG, Xie YN, Zhang XY, Gao YL. Coping styles and personality characteristics of naval officers and soldiers in the Southern Theater Command. Chin Ment Health J. 2004;18(3):179. 10.3321/j.issn:1000-6729.2004.03.011.

[32] Xie YN. Reliability and validity of the Simplified Coping Style Questionnaire. Chin J Clin Psychol. 1998;6(2):53–4. 10.16128/j.cnki.1005-3611.1998.02.018.

[33] Chen F, Liu G, Mair CA. Intergenerational Ties in Context: Grandparents Caring for Grandchildren in China. Soc Forces. 2011;90(2):571–94. 10.1093/sf/sor012. 22544978 PMC3337769

[34] Zhu Z, Wang Y, Wang X, Zhang F, Yu L, Liu T. The independent and joint role of socioeconomic status and family relationships on mortality risk in China: cultural differences and health inequalities in the context of intergenerational cohabitation. Arch Public Health. 2025;83(1):187. 10.1186/s13690-025-01669-2. Published 2025 Jul 15. 40660323 PMC12261701

[35] Chen F, Shen K, Ruan H. The mixed blessing of living together or close by: Parent-child relationship quality and life satisfaction of older adults in China. Demogr Res. 2021;44:563–94. 10.4054/demres.2021.44.24. 34054340 PMC8153673

[36] Jakicic JM, Apovian CM, Barr-Anderson DJ, et al. Physical Activity and Excess Body Weight and Adiposity for Adults. American College of Sports Medicine Consensus Statement. Med Sci Sports Exerc. 2024;56(10):2076–91. 10.1249/MSS.0000000000003520. 39277776

[37] Su X, Zhang Y, Chen M, Wang H, Liu G. Influencing factors and risk prediction modeling of maternal postpartum depression: a cross-sectional study in Chinese puerperal women of sitting the month. Front Psychiatry. 2023;14:1252789. 10.3389/fpsyt.2023.1252789. Published 2023 Sep 14. 37779623 PMC10539603

[38] Linde K, Lehnig F, Nagl M, Stepan H, Kersting A. Course and prediction of body image dissatisfaction during pregnancy: a prospective study. BMC Pregnancy Childbirth. 2022;22(1):719. 10.1186/s12884-022-05050-x. Published 2022 Sep 20. 36127633 PMC9487034

[39] Watson B, Fuller-Tyszkiewicz M, Broadbent J, Skouteris H. The meaning of body image experiences during the perinatal period: A systematic review of the qualitative literature. Body Image. 2015;14:102–13. 10.1016/j.bodyim.2015.04.005. 25950953

[40] Cho H, Lee K, Choi E, et al. Association between social support and postpartum depression. Sci Rep. 2022;12(1):3128. 10.1038/s41598-022-07248-7. Published 2022 Feb 24. 35210553 PMC8873474

[41] Pollet TV, Dawson S, Tovée MJ, Cornelissen PL, Cornelissen KK. Fat talk is predicted by body dissatisfaction and social comparison with no interaction effect: Evidence from two replication studies. Body Image. 2021;38:317–24. 10.1016/j.bodyim.2021.05.005. 34087541

[42] Soyturk A, Egelioglu Cetisli N. Eating attitude and body satisfaction of adolescent and non-adolescent pregnant women. Rev Assoc Med Bras (1992). 2025;71(2):e20241184. Published 2025 Mar 31. 10.1590/1806-9282.20241184 PMC11964308 40172390

