# Supplementary material for: A study on current status and influencing factors of pregnant women’s body image in China: A mixed-methods study
Source: BMC Pregnancy Childbirth. 2026 Jun 26;26:1008. doi: 10.1186/s12884-026-09463-w (PMC13563722; doi:10.1186/s12884-026-09463-w)
Supplement: Supplementary file 1 [file 12884_2026_9463_MOESM1_ESM.docx]

Body Image Score of Pregnant Women (n=160)

| Dimension | Number Of Entries | Score range |  |
| --- | --- | --- | --- |
| Discontent With Body Parts | 7 | 7～31 | 19.80±5.29 |
| Physical Dissatisfaction | 5 | 5～23 | 12.18±4.06 |
| Appearance Attention | 4 | 5～20 | 12.82±3.34 |
| Discontent With Facial Features | 4 | 4～20 | 10.13±2.93 |
| Attraction To The Opposite Sex | 4 | 6～18 | 11.18±1.90 |
| Controlling Weight Due To Appearance | 3 | 2～10 | 6.49±1.71 |
| Appearance Is Superior To Physical Function | 3 | 3～13 | 9.66±2.18 |
| Discontent With Physiological Changes During Pregnancy | 2 | 2～10 | 5.19±1.81 |
| Appearance Related Avoidance Behavior | 3 | 2～10 | 5.77±1.87 |
| Total Score Of Maternal Body Imagery | 35 | 53～147 | 97.78±15.57 |

Pregnant women's perception of social support score（n=160）

| Dimension | Number Of Entries | Score range |  |
| --- | --- | --- | --- |
| Family Support | 4 | 12～28 | 24.02±3.63 |
| Friend Support | 4 | 4～28 | 22.12±4.25 |
| Other Support | 4 | 4～28 | 20.24±4.87 |
| Total Score Of Understanding Social Support | 12 | 40～84 | 66.39±10.84 |

Pregnant women's understanding of social support levels（n=160）

| Pregnant Women's Understanding Of Social Support Levels | Frequency |
| --- | --- |
| Secondary | 31.3% |
| Higher | 68.8% |
