# Supplementary material for: A study on current status and influencing factors of pregnant women’s body image in China: A mixed-methods study
Source: BMC Pregnancy Childbirth. 2026 Jun 26;26:1008. doi: 10.1186/s12884-026-09463-w (PMC13563722; doi:10.1186/s12884-026-09463-w)
Supplement: Supplementary file 2 [file 12884_2026_9463_MOESM2_ESM.docx]

**Detailed analytical procedure**

Below, we provide a detailed, step-by-step account of our analytical procedure, adhering to the six-phase framework of reflexive thematic analysis by Braun and Clarke, which guided our study:

1. Data Immersion and Initial Noting:

All audio recordings were transcribed verbatim by the first author (Y.F.Z.), resulting in approximately 100,000 words of textual data.

The research team (Y.F.Z. and R.Y.) repeatedly read the transcripts independently to achieve deep familiarity. During this phase, we wrote preliminary analytic memos on initial impressions, potential patterns, and contextual observations.

2. Generating Initial Codes:

We began systematic coding, working in a data-driven (inductive) manner. Codes were assigned to segments of text that captured a specific idea, feeling, or experience relevant to the research question (e.g., “husband’s reassurance,” “mother-in-law’s criticism on weight,” “feeling of loss of control over body”).

Coding was conducted iteratively. The first two authors coded several transcripts independently, then met to compare, discuss, and refine the codebook to ensure consistency. This process involved merging similar codes, splitting broad ones, and clarifying definitions.

3. Searching for Themes:

We then examined all codes and collated them into potential candidate themes. This involved asking: “What broader pattern of meaning do these codes collectively suggest?” We grouped codes visually to identify conceptual clusters (e.g., grouping codes related to family interactions under a potential theme of "Social Support as Cultural Validation/Surveillance").

4. Reviewing and Refining Themes:

This was a crucial, recursive phase. We reviewed the candidate themes against two levels:

a) Level 1: Reviewing themes in relation to the coded extracts. We checked if all data within a theme cohered meaningfully, and if the theme accurately represented the coded data.

b) Level 2: Reviewing themes in relation to the entire dataset. We re-read the full transcripts to ensure themes captured the most significant and prevalent patterns across all participants, and that we had not forced data into themes.

Themes were refined, split, combined, or discarded during this process. For instance, an initial broad theme “Family Influence” was differentiated into the more nuanced “Social Support from Different Sources” and “Desire for Recognition.”

5. Defining and Naming Themes:

For each finalized theme, we wrote a detailed analytical definition that captured its essence and scope. We then named themes to convey their core meaning in a concise and evocative way (e.g., “Emotional Coping Styles”).

We ensured that themes were not just summaries of data, but represented interpretive arguments about the data within the Chinese sociocultural context.

6. Producing the Report:

The final analytic write-up (as seen in the Results section) was developed. We selected vivid, compelling, and representative extracts from the transcripts to embed within the narrative for each theme, ensuring a clear link between the presented evidence and our analytic claims.
