# Supplementary material for: A study on current status and influencing factors of pregnant women’s body image in China: A mixed-methods study
Source: BMC Pregnancy Childbirth. 2026 Jun 26;26:1008. doi: 10.1186/s12884-026-09463-w (PMC13563722; doi:10.1186/s12884-026-09463-w)
Supplement: Supplementary file 3 [file 12884_2026_9463_MOESM3_ESM.docx]

***Demography data and Obstetric data***

***1.sociodemographic characteristics questionnaire***

Thank you very much for your support of this study! Please check (√) the option that best fits your personal situation or fill in the corresponding content on the line.

1.Your Ethnicity ______ A. Han Chinese B. Ethnic Minority ______ (Specify)

2.Your Age ______ (years old)

3.Height ______ (meters)

4.Waist Circumference ______ (centimeters)

5.Current Weight ______ (kilograms)

6.Pre-pregnancy Weight ______ (kilograms)

7.Do you have a religious belief? A. No B. Yes ______ (Specify)

8.Your Education Level______

A. Primary School

B. Junior High School

C. Senior High School or Technical Secondary School

D. College (Associate Degree)

E. Undergraduate (Bachelor's Degree)

F. Postgraduate (Master's/Doctoral Degree)

9.Your Family Locations ______ A. Urban B. Rural

10.Your Occupations ______

A. Civil Servant

B. Teacher

C. Worker

D. Company Employee

E. Business Owner

F. Farmer

G. Homemaker

H. Other (Please Specify) ______

11.Your Family's Average Monthly Income per Person (RMB)s ______

A. Below 5,000

B. 5,000 - 10,000

C. 10,000 - 15,000

D. Above 15,000

12.Who do you live with?

A. Spouse

B. Grandparents (elder generation) ______

C. Friends

D. No one (Alone)

E. Other ______(Please Specify)

13.How is your relationship with your husband?

A. Very Close

B. Relatively Close

C. Average

D. Poor, Frequent Arguments

14.What do you think about your own weight?

A. Severely Underweight

B. Underweight

C. Normal

D. Overweight

E. Severely Overweight

15.What do your family and friends think about your weight?

A. Severely Underweight

B. Underweight

C. Normal

D. Overweight

E. Severely Overweight

16.Gestational Weeks: ______

17.Gravidity (including this pregnancy): ______

18.Parity: ______

19.Pregnancy Intention: ______

20.History of Abortion: A. Yes B. No

21.Did you use Assisted Reproductive Technology (e.g., IVF) for this pregnancy? A. Yes B. No

22.How much time per day do you spend learning about pregnancy-related knowledge from mass media (e.g., TV, magazines, radio, internet, mobile phone, etc.)?

A. Less than 1 hour/day

B. 1~3 hours/day

C. 3~5 hours/day

D. More than 5 hours/day

23.To what extent do you think mass media influences your perception of your body shape and weight during pregnancy?

A. Not influenced at all

B. Hardly influenced

C. Somewhat influenced

D. Moderately influenced

E. Greatly influenced

***Body Image in Pregnancy***

***2.Body image in Pregnancy Scale (BIPS)（Chinese Ver.）***

The following statements are about your physical aspects during pregnancy. Please recall your current situation (over the past two weeks). If you strongly disagree with the statement, please choose 1; if you disagree, choose 2; if you neither agree nor disagree, choose 3; if you agree, choose 4; if you strongly agree, choose 5.

|  | 1.Strongly Disagree | 2.Disagree | 3.Neither Agree nor Disagree | 4.Agree | 5.Strongly Agree |
| --- | --- | --- | --- | --- | --- |
| 1. I spend a lot of time thinking about my weight during pregnancy. |  |  |  |  |  |
| 2. I spend a lot of time thinking about my body shape during pregnancy. |  |  |  |  |  |
| 3. I spend a lot of time thinking about my physical appearance during pregnancy. |  |  |  |  |  |
| 4. I very much desire a slim body shape during pregnancy. |  |  |  |  |  |
| 5. During pregnancy, I am reluctant to let my partner see my naked pregnant body. |  |  |  |  |  |
| 6. During pregnancy, I worry that my body is not attractive to my partner. |  |  |  |  |  |
| 7. During pregnancy, I believe sexual satisfaction is related to bodily changes. |  |  |  |  |  |
| 8. During pregnancy, I think more about maternal and infant health than about physical appearance. |  |  |  |  |  |
| 9. During pregnancy, I think more about what I can do than about physical appearance. |  |  |  |  |  |
| 10. During pregnancy, I think more about the function of my breasts than about their appearance. |  |  |  |  |  |
| 11. I would stop breastfeeding early or not breastfeed at all due to concerns about sagging breasts. |  |  |  |  |  |

The following questions are about your feelings regarding bodily changes during pregnancy. Please recall your current situation (over the past two weeks). If you are very satisfied, choose 1; if you are basically satisfied, choose 2; if you are neither satisfied nor dissatisfied, choose 3; if you are dissatisfied, choose 4; if you are very dissatisfied, choose 5.

|  | 1. Very Satisfied | 2. Basically Satisfied | 3. Neither Satisfied nor Dissatisfied | 4. Dissatisfied | 5.VeryDissatisfied |
| --- | --- | --- | --- | --- | --- |
| 12. During pregnancy, are you satisfied with your body flexibility? |  |  |  |  |  |
| 13. During pregnancy, are you satisfied with your physical strength? |  |  |  |  |  |
| 14. During pregnancy, are you satisfied with your energy levels? |  |  |  |  |  |
| 15. Are you satisfied with the changes in body flexibility caused by pregnancy? |  |  |  |  |  |
| 16. Are you satisfied with the changes in physical strength caused by pregnancy? |  |  |  |  |  |
| 17. During pregnancy, are you satisfied with the complexion of your face? |  |  |  |  |  |
| 18. During pregnancy, are you satisfied with your hair (density, texture)? |  |  |  |  |  |
| 19. Are you satisfied with the changes in facial complexion caused by pregnancy? |  |  |  |  |  |
| 20. Are you satisfied with the changes in your hair (density, texture) caused by pregnancy? |  |  |  |  |  |
| 21. During pregnancy, are you satisfied with your chest? |  |  |  |  |  |
| 22. During pregnancy, are you satisfied with the width of your shoulders? |  |  |  |  |  |
| 23. During pregnancy, are you satisfied with your ankles? |  |  |  |  |  |
| 24. During pregnancy, are you satisfied with your arms? |  |  |  |  |  |
| 25. During pregnancy, are you satisfied with your hands and feet? |  |  |  |  |  |
| 26. During pregnancy, are you satisfied with your hips? |  |  |  |  |  |
| 27. During pregnancy, are you satisfied with your legs? |  |  |  |  |  |
| 28. During pregnancy, are you satisfied with the swelling (edema) you experience? |  |  |  |  |  |
| 29. During pregnancy, are you satisfied with your stretch marks? |  |  |  |  |  |

The following are questions about your own behaviors during pregnancy. Please recall your current situation (over the past two weeks). If you have never engaged in this behavior, choose 1; if you rarely have, choose 2; if you occasionally have, choose 3; if you frequently have, choose 4; if you always have, choose 5.

|  | 1. Never | 2. Rarely | 3.Occasionally | 4.Frequently | 5. Always |
| --- | --- | --- | --- | --- | --- |
| 30. Do you avoid exercise due to feeling physically unstable during pregnancy? |  |  |  |  |  |
| 31. During pregnancy, does dissatisfaction with your physical appearance prevent you from participating in social activities (e.g., gatherings)? |  |  |  |  |  |
| 32. During pregnancy, do you restrict your diet because you want to maintain your figure? |  |  |  |  |  |
| 33. During pregnancy, do you feel the need to exercise because you are concerned about changes in your body shape? |  |  |  |  |  |
| 34. During pregnancy, do you feel the need to exercise because you are concerned about changes in your weight? |  |  |  |  |  |
| 35. During pregnancy, do you refuse sexual activity because of bodily changes? |  |  |  |  |  |

***Perceived Social Support***

***3.The Perceived Social Support Scale (PSSS)***

The following statements are about the social support you feel during your pregnancy. Please think about your current situation over the past two weeks. If you strongly disagree with a statement, choose 1; if you disagree, choose 2; if you somewhat disagree, choose 3; if you are neutral, choose 4; if you somewhat agree, choose 5; if you agree, choose 6; if you strongly agree, choose 7.

|  | 1.Strongly Disagree | 2.Disagree | 3.Somewhat Disagree | 4.Neutral | 5. Somewhat Agree | 6.Agree | 7.Strongly Agree |
| --- | --- | --- | --- | --- | --- | --- | --- |
| 1.When I encounter problems, there are people (superiors, neighbors, and community members) by my side. |  |  |  |  |  |  |  |
| 2.I can share my joys and sorrows with some people (superiors, neighbors, and community members). |  |  |  |  |  |  |  |
| 3.My family can provide me with tangible and concrete help. |  |  |  |  |  |  |  |
| 4.When needed, I can obtain emotional help and support from my family. |  |  |  |  |  |  |  |
| 5.When I'm in difficulty, some people (superiors, neighbors, and community members) are a true source of comfort for me. |  |  |  |  |  |  |  |
| 6.My friends can truly help me. |  |  |  |  |  |  |  |
| 7.In times of trouble, I can rely on my friends. |  |  |  |  |  |  |  |
| 8.I can discuss my problems with my family. |  |  |  |  |  |  |  |
| 9.My friends can share my joys and sorrows. |  |  |  |  |  |  |  |
| 10.In my life, there are people (superiors, neighbors, and community members) who care about my feelings. |  |  |  |  |  |  |  |
| 11.My family can help me make various decisions. |  |  |  |  |  |  |  |
| 12.I can discuss my problems with my friends. |  |  |  |  |  |  |  |

***Coping Style***

***4.Simplified Coping Style Questionnaire (SCSQ)***

The following list presents possible attitudes and strategies you might adopt when encountering setbacks, difficulties, or challenges in life. Please read each item carefully and select the response that best reflects your personal experience by checking the corresponding box in the answer column. The options are:

|  | 0.Never | 1.Seldom | 2.Sometimes | 3.Often | 4.Always |
| --- | --- | --- | --- | --- | --- |
| 1.Divert attention through work, study, or other activities. |  |  |  |  |  |
| 2.Change my way of thinking, rediscovering what is important in life. |  |  |  |  |  |
| 3.Try to see the positive side of things. |  |  |  |  |  |
| 4.Talk to someone and pour out my inner troubles. |  |  |  |  |  |
| 5.Try not to take the problem too seriously. |  |  |  |  |  |
| 6.Stand my ground and strive for what I want. |  |  |  |  |  |
| 7.Find several different ways to solve the problem. |  |  |  |  |  |
| 8.Seek advice from relatives, friends, or classmates. |  |  |  |  |  |
| 9.Change my original approach or address some of my own issues. |  |  |  |  |  |
| 10.Learn from how others have handled similar difficult situations. |  |  |  |  |  |
| 11.Take up a hobby or actively participate in recreational activities. |  |  |  |  |  |
| 12.Try to restrain feelings of disappointment, regret, sadness, or anger. |  |  |  |  |  |
| 13.Try to rest or take a vacation to temporarily set the problem aside. |  |  |  |  |  |
| 14.Use smoking, drinking, taking medication, or eating to relieve distress. |  |  |  |  |  |
| 15.Believe time will change the situation, thinking that the only thing to do is wait. |  |  |  |  |  |
| 16.Try to forget the whole thing. |  |  |  |  |  |
| 17.Depend on others to solve the problem. |  |  |  |  |  |
| 18.Accept reality because there is no other option. |  |  |  |  |  |
| 19.Fantasize that some miracle might happen to change the situation. |  |  |  |  |  |
| 20.Console myself. |  |  |  |  |  |
