# Supplementary material for: A study on current status and influencing factors of pregnant women’s body image in China: A mixed-methods study
Source: BMC Pregnancy Childbirth. 2026 Jun 26;26:1008. doi: 10.1186/s12884-026-09463-w (PMC13563722; doi:10.1186/s12884-026-09463-w)
Supplement: Supplementary file 4 [file 12884_2026_9463_MOESM4_ESM.docx]

**Interview Guide**

**Opening Statement**

Hello, my name is Yi Feizhang. I am a researcher from Chinese PLA General Hospital, and I am part of the research team conducting this study.

Thank you very much for agreeing to talk with us today. Before we begin, I would like to take a few moments to explain the purpose of this interview and how the information you share will be used.

We are conducting this study to understand the experiences of pregnant women regarding their body image—specifically, how you feel about the changes in your body during pregnancy, and what factors may influence these feelings. Your perspective is incredibly valuable, as it will help us better understand the needs of pregnant women in China and inform future healthcare support.

A few important points before we start:

Voluntary Participation: Your participation in this interview is completely voluntary. You may choose not to answer any question, and you are free to stop the interview at any time without giving any reason. This will not affect your medical care or any services you receive.

Confidentiality: Everything you share with us today will be kept strictly confidential. Your name and any identifying information will be removed from all records and replaced with a code. In any reports or publications, we will use pseudonyms to ensure that you cannot be identified.

Recording: With your permission, I would like to audio-record this interview. The recording will help me accurately capture your thoughts and experiences. The recording will only be accessible to the research team and will be destroyed after the study is completed.

Duration: The interview will take approximately 15–30 minutes.

Do you have any questions before we begin? If you are comfortable and agree to participate, we will start now.

**Interview Questions**

**Question 1 (Open‑ended introduction):**

“Since becoming pregnant, what noticeable feelings or thoughts have you had about the changes in your body—such as weight, shape, skin, or other aspects? Could you share some specific examples?”

**Question 2 (Family and social interactions):**

“Have people around you—family, friends, or colleagues—commented on or paid attention to your bodily changes? How have these reactions affected your mood or how you see yourself?”

**Question 3 (Coping and emotions):**

“When you experience emotions or stress related to bodily changes, what do you usually do? Are there any habits or strategies that help you adjust to these changes?”

**Question 4 (Cultural norms and self‑expectations):**

“In your view, what are the societal or traditional expectations about ‘how a pregnant woman should look or be’? How do these ideas influence the way you perceive your own body?”

**Question 5 (Support needs and suggestions):**

“What kind of support or information would you like to receive from healthcare providers, family, or society to help you better accept your body during pregnancy?”
